# Supplementary material for: Short-Wave Infrared Hyperspectral Image-Based Quality Grading of Dried Laver (Pyropia spp.)
Source: Foods. 2025 Feb 4;14(3):497. doi: 10.3390/foods14030497 (PMC11817384; doi:10.3390/foods14030497)
Supplement: Supplementary file 1 [file foods-14-00497-s001.zip › foods-3444764-supplementary.pdf]

Supplementary Material

Table S1. Description of sample information of dried laver

Table S2. Proximate composition (g/100 g) of dried laver

Table S3. ATP-related compound content (g/100 g) of dried laver

Table S4. Organic acid content (g/100 g) of dried laver

Table S5. Total amino acid content (g/100 g) of dried laver

Table S6. Cutting stress (kg·mm) of dried laver

Table S7. Accuracy and R^2 value of PLS-DA class 1 in quality index

Table S8. Accuracy and R^2 value of PLS-DA class 2 in quality index

Table S9. Accuracy and R^2 value of PLS-DA class 3 in quality index

Table S10. Classification performance of each regression model

Table S1. Description of sample information of dried laver

Sample No.	Product type	origin	species	Size (mm)	Pixel
1	GG[1)]	Goheung-gun	*Pyropia yezoensis*	Length: 210 ± 5<br>Width: 190 ±5	315×300
2	GG	Wando-gun	*Pyropia yezoensis*	Length: 210 ± 5<br>Width: 190 ±5	315×300
3	GG	Wando-gun	*Pyropia yezoensis*	Length: 210 ± 5<br>Width: 190 ±5	315×300
4	GG	Wando-gun	*Pyropia yezoensis*	Length: 210 ± 5<br>Width: 190 ±5	315×300
5	GG	Wando-gun	*Pyropia yezoensis*	Length: 210 ± 5<br>Width: 190 ±5	315×300
6	DG[2)]	Muan-gun	*Pyropia seriata,*<br>*Porphyra dentata kjellman*	Length: 265 ± 5<br>Width: 190 ±5	400×300
7	DG	Jindo-gun	*Pyropia seriata,*<br>*Porphyra dentata kjellman*	Length: 265 ± 5<br>Width: 190 ±5	400×300
8	DG	Haenam-gun	*Pyropia seriata,*<br>*Porphyra dentata kjellman*	Length: 265 ± 5<br>Width: 190 ±5	400×300
9	JG[3)]	Haenam-gun	*Pyropia yezoensis*	Length: 265 ± 5<br>Width: 190 ±5	400×300
10	JG	Haenam-gun	*Pyropia yezoensis*	Length: 265 ± 5<br>Width: 190 ±5	400×300
11	DG	Haenam-gun	*Pyropia seriata,*<br>*Porphyra dentata kjellman*	Length: 265 ± 5<br>Width: 190 ±5	400×300
12	DG	Haenam-gun	*Pyropia seriata,*<br>*Porphyra dentata kjellman*	Length: 265 ± 5<br>Width: 190 ±5	400×300
13	JG	Haenam-gun	*Pyropia yezoensis*	Length: 265 ± 5<br>Width: 190 ±5	400×300
14	GG	Haenam-gun	*Pyropia yezoensis*	Length: 210 ± 5<br>Width: 190 ±5	315×300
15	GG	Haenam-gun	*Pyropia yezoensis*	Length: 210 ± 5<br>Width: 190 ±5	315×300
16	DG	Seocheon-gun	*Pyropia seriata,*<br>*Porphyra dentata kjellman*	Length: 265 ± 5<br>Width: 190 ±5	400×300
17	JG	Seocheon-gun	*Pyropia yezoensis*	Length: 265 ± 5<br>Width: 190 ±5	400×300
18	JG	Seocheon-gun	*Pyropia yezoensis*	Length: 265 ± 5<br>Width: 190 ±5	400×300
19	GG	Seocheon-gun	*Pyropia yezoensis*	Length: 210 ± 5<br>Width: 190 ±5	315×300
20	DG	Shinan-gun	*Pyropia seriata,*<br>*Porphyra dentata kjellman*	Length: 265 ± 5<br>Width: 190 ±5	400×300
21	DG	Shinan-gun	*Pyropia seriata,*	Length: 265 ± 5	400×300

			Porphyra dentata kjellman	Width: 190 ±5	
22	DG	Busan	*Pyropia seriata, Porphyra dentata kjellman*	Length: 265 ± 5<br>Width: 190 ±5	400×300
23	JG	Busan	*Pyropia yezoensis*	Length: 265 ± 5<br>Width: 190 ±5	400×300
24	JG	Busan	*Pyropia yezoensis*	Length: 265 ± 5<br>Width: 190 ±5	400×300
25	JG	Busan	*Pyropia yezoensis*	Length: 265 ± 5<br>Width: 190 ±5	400×300

[1)]Dried laver, Jaelae Gim (JG), is the product of raw laver, which belongs to Pyropia yezoensis etc. [2)]Dried laver, Dol Gim (DG), is the product of raw laver, which belongs to P. dentata kjellman and P. seriata. [3)]Dried laver, Gimbab Gim (GG), is the product to make Korean rice rolls, which belongs to P. yezoensis

Table S2. Proximate composition (g/100 g) of dried laver

Sample	Moisture	Ash	Crude protein	Crude lipids
1	9.883±0.238$^{cde1)}$	8.667±0.010^{i}	41.230±0.239^{c}	0.222±0.003^{klm}
2	9.497±0.197^{efg}	8.845±0.173^{ghi}	44.190±0.000^{a}	0.203±0.003^{lm}
3	9.676±0.222^{ef}	9.211±0.078^{def}	41.060±0.000^{cd}	0.577±0.015^{cde}
4	9.414±0.230^{efg}	9.472±0.031^{cd}	40.107±0.033^{defg}	0.600±0.002^{cde}
5	7.584±0.148^{k}	10.147±0.065^{b}	40.957±0.434^{cd}	0.619±0.020^{cd}
6	9.113±0.099^{fgh}	8.856±0.029^{ghi}	39.087±0.107^{h}	0.348±0.008^{ghij}
7	9.965±0.201^{cde}	10.441±0.051^{a}	34.377±0.053^{m}	0.671±0.032^{c}
8	9.997±0.236^{cde}	8.314±0.094^{j}	32.273±0.282^{n}	0.374±0.013^{gh}
9	8.718±0.319^{hij}	6.738±0.059^{n}	39.813±0.233^{efgh}	0.399±0.002^{gh}
10	8.611±0.102^{hij}	7.651±0.037^{m}	39.400±0.229^{fgh}	0.620±0.011^{cd}
11	8.288±0.213^{j}	9.047±0.049^{efg}	36.243±0.181^{jk}	0.600±0.035^{cde}
12	8.469±0.268^{ij}	7.654±0.013^{m}	37.860±0.162^{i}	0.440±0.000^{fg}
13	9.020±0.010^{ghi}	9.603±0.004^{c}	39.220±0.238^{fgh}	0.524±0.042^{def}
14	9.542±0.021^{efg}	7.636±0.039^{m}	39.162±0.153^{gh}	0.130±0.008^{m}
15	6.071±0.042^{l}	9.300±0.165^{de}	42.755±0.204^{b}	0.267±0.008^{ijkl}
16	12.038±0.027^{a}	7.829±0.044^{lm}	36.578±0.065^{j}	0.133±0.009^{m}
17	10.032±0.019^{cde}	10.428±0.101^{a}	44.987±0.058^{a}	0.169±0.008^{lm}
18	9.956±0.089^{cde}	10.571±0.034^{a}	40.156±0.860^{def}	0.324±0.012^{hijk}
19	9.935±0.045^{cde}	10.378±0.063^{ab}	40.691±0.144^{cde}	0.508±0.033^{ef}
20	11.050±0.111^{b}	7.791±0.031^{lm}	35.812±0.097^{jkl}	0.828±0.056^{b}
21	10.482±0.139^{bc}	8.743±0.121^{hi}	35.115±0.185^{lm}	0.258±0.000^{jkl}
22	9.763±0.155^{de}	7.972±0.035^{kl}	35.830±0.033^{jkl}	0.369±0.024^{ghi}
23	9.797±0.141^{de}	9.141±0.046^{ef}	41.326±0.110^{c}	1.508±0.051^{a}
24	7.221±0.026^{k}	8.140±0.025^{jk}	41.285±0.073^{c}	1.525±0.071^{a}
25	10.318±0.165^{cd}	9.009±0.065^{fgh}	35.474±0.318^{kl}	0.657±0.016^{c}

[1)] The different letters on the data in the column indicate significant differences at $P<0.05$.

Table S3. ATP-related compound content (g/100 g) of dried laver

Sample	ATP	ADP	AMP	IMP
1	25.826±0.279[bc1)]	55.684±0.549[defg]	111.748±1.002[c]	4.728±2.047[a]
2	23.331±0.316[bcde]	63.540±1.673[abc]	116.296±0.919[bc]	1.761±0.038[cde]
3	23.500±0.192[bcde]	54.797±0.659[efg]	126.698±4.050[abc]	1.078±0.021[de]
4	14.125±0.694[hi]	42.592±1.386[ijk]	119.255±10.109[bc]	1.847±0.793[cde]
5	26.307±0.846[b]	52.714±1.251[fgh]	141.231±5.684[a]	1.815±0.311[cde]
6	32.207±1.232[a]	63.245±1.523[abcd]	84.835±3.365[d]	1.290±0.061[cde]
7	10.023±0.403[j]	33.728±0.343[lm]	67.421±6.049[e]	1.192±0.025[de]
8	18.089±0.526[fg]	49.924±0.685[ghij]	64.565±14.293[e]	3.688±0.113[ab]
9	16.477±0.629[gh]	46.611±1.613[hij]	122.171±4.684[bc]	0.476±0.012[e]
10	22.406±0.575[de]	50.425±0.843[ghi]	132.046±3.994[ab]	1.779±0.086[cde]
11	16.310±0.003[gh]	67.636±2.121[a]	12.172±0.230[g]	2.998±0.315[abc]
12	15.729±0.674[ghi]	61.991±2.667[abcde]	11.803±0.502[g]	1.630±0.046[cde]
13	20.687±0.773[ef]	66.961±1.871[a]	22.521±0.703[fg]	0.713±0.061[de]
14	12.622±0.435[ij]	46.051±0.417h[ij]	20.665±0.132[fg]	0.882±0.050[de]
15	5.937±0.283[k]	27.632±0.691[m]	33.742±0.764[f]	1.077±0.086[de]
16	13.127±0.809[hij]	34.401±1.228lm	19.785±1.350[fg]	0.104±0.022[e]
17	13.867±0.137[hi]	68.308±3.642[a]	26.272±1.072[fg]	1.321±0.143[cde]
18	25.523±0.872[bcd]	64.915±0.670[ab]	19.755±0.281[fg]	2.283±0.279[bcd]
19	21.826±0.135[e]	53.293±4.051[fgh]	26.042±1.674[fg]	0.763±0.033[de]
20	22.886±1.399[cde]	54.856±3.068[efg]	12.425±0.570[g]	0.732±0.015[de]
21	20.498±0.755[ef]	42.495±3.306[jk]	17.922±0.119[fg]	0.585±0.009[de]
22	26.206±0.065[bc]	58.791±3.265[bcdef]	23.372±1.637[fg]	1.689±0.081[cde]
23	26.405±2.796[b]	63.371±0.204[abcd]	21.903±1.194[fg]	1.143±0.030[de]
24	10.134±0.839[j]	36.620±0.636[kl]	23.529±0.562[fg]	1.739±0.025[cde]
25	15.540±1.080[ghi]	56.489±3.245[cdefg]	16.882±0.321[g]	0.743±0.001[de]

[1)] The different letters on the data in the column indicate significant differences at $P<0.05$.

Table S3. (Continued)

Sample	Inosine	Hypoxanthine	GMP	Guanosine
1	ND	ND	6.413±0.092^{ghij}	2.476±0.076^{c}
2	ND	ND	7.013±0.141^{ghij}	2.608±0.052^{bc}
3	ND	ND	8.208±0.048^{ghij}	2.881±0.089^{ab}
4	ND	ND	10.621±0.054^{ghi}	2.780±0.043^{abc}
5	ND	ND	6.200±1.591^{ghij}	2.893±0.074^{ab}
6	ND	ND	4.183±0.344^{j}	2.005±0.095^{d}
7	ND	ND	4.919±0.121^{ij}	2.800±0.233^{abc}
8	ND	ND	5.889±0.301^{hij}	2.964±0.082^{a}
9	ND	ND	9.026±0.037^{ghij}	1.621±0.004^{e}
10	ND	ND	8.911±0.408^{ghij}	1.618±0.001^{e}
11	3.531±0.138^{f}[1)]	1.844±0.000^{abc}	79.833±2.319^{a}	ND
12	2.895±0.074^{i}	1.808±0.048^{bcd}	38.689±2.092^{cd}	ND
13	3.285±0.011^{fgh}	1.762±0.017^{cd}	33.593±1.128^{de}	ND
14	4.364±0.046^{bc}	1.820±0.015^{bcd}	36.090±0.854^{cde}	ND
15	4.730±0.236^{ab}	1.797±0.024^{bcd}	31.821±0.763^{e}	ND
16	3.609±0.042^{ef}	1.910±0.010^{ab}	10.554±0.156^{ghi}	ND
17	4.813±0.173^{a}	1.846±0.031^{abc}	19.975±0.780^{f}	ND
18	3.411±0.064^{fg}	1.835±0.028^{abc}	44.805±1.216^{b}	ND
19	4.273±0.072^{cd}	1.767±0.049^{cd}	40.846±5.979^{bc}	ND
20	4.360±0.008^{c}	1.948±0.054^{a}	11.031±0.637^{gh}	ND
21	2.918±0.115^{i}	1.833±0.041^{abc}	20.158±0.057^{f}	ND
22	2.985±0.065^{hi}	1.809±0.025^{bcd}	11.804±0.104^{g}	ND
23	3.971±0.068^{de}	1.821±0.013^{bcd}	35.799±0.475^{cde}	ND
24	3.587±0.036^{f}	1.712±0.044^{d}	34.585±0.363^{de}	ND
25	3.153±0.033^{ghi}	1.813±0.033^{bcd}	20.964±0.720^{f}	ND

[1)] The different letters on the data in the column indicate significant differences at $P<0.05$.

Table S4. Organic acid content (g/100 g) of dried laver

Sample	Oxalic acid	Citric acid	Malic acid	Maleic acid
1	0.083±0.000[hij1)]	0.052±0.001[l]	0.146±0.008[fg]	0.003±0.000[fg]
2	0.112±0.002[def]	0.143±0.006[efg]	0.123±0.013[gh]	0.002±0.000[fg]
3	0.017±0.000[m]	0.100±0.002[hijk]	0.056±0.002[ij]	0.002±0.000[fg]
4	0.108±0.003[efg]	0.084±0.005[ijkl]	0.066±0.004[hij]	0.003±0.000[fg]
5	0.071±0.001[ijk]	0.079±0.006[jkl]	0.051±0.001[j]	0.002±0.000[fg]
6	0.046±0.005[l]	0.232±0.002[ab]	0.249±0.001[cde]	0.002±0.000[fg]
7	0.055±0.002[kl]	0.116±0.002[ghij]	0.016±0.003[j]	0.003±0.000[fg]
8	0.057±0.003[kl]	0.191±0.000[cd]	0.050±0.000[j]	0.002±0.000[fg]
9	0.059±0.001[jkl]	0.119±0.001[ghi]	0.111±0.005[ghi]	0.002±0.000[fg]
10	0.088±0.003[ghi]	0.099±0.003[hijk]	0.161±0.005[fg]	0.001±0.000[fg]
11	0.087±0.002[ghi]	0.261±0.030[a]	0.307±0.008[abc]	0.271±0.023[ab]
12	0.018±0.001[m]	0.126±0.004[gh]	0.331±0.013[ab]	0.001±0.000[g]
13	0.079±0.001[hij]	0.097±0.009[hijk]	0.281±0.021[bcd]	0.171±0.001[cd]
14	0.108±0.015[efg]	0.201±0.009[bcd]	0.241±0.009[de]	0.217±0.003[bc]
15	0.220±0.002[b]	0.179±0.005[de]	0.328±0.009[ab]	0.220±0.040[bc]
16	0.024±0.005[m]	0.072±0.006[kl]	0.152±0.008[fg]	0.194±0.001[cd]
17	0.240±0.004[a]	0.150±0.012[efg]	0.254±0.025[cde]	0.057±0.006[ef]
18	0.089±0.002[fghi]	0.170±0.004[def]	0.205±0.005[ef]	0.182±0.010[cd]
19	0.071±0.001[ijk]	0.179±0.019[de]	0.254±0.006[cde]	0.250±0.010[ab]
20	0.074±0.002[ijk]	0.067±0.009[kl]	0.261±0.048[cde]	0.301±0.038[a]
21	0.097±0.002[fgh]	0.073±0.007[kl]	0.227±0.025[de]	0.076±0.006[e]
22	0.093±0.007[fghi]	0.138±0.006[fg]	0.261±0.007[cde]	0.072±0.006[e]
23	0.131±0.014[cd]	0.102±0.014[hijk]	0.344±0.024[a]	0.029±0.004[efg]
24	0.139±0.004[c]	0.225±0.008[bc]	0.362±0.006[a]	0.169±0.024[cd]
25	0.129±0.007[cde]	0.230±0.005[ab]	0.244±0.008[de]	0.157±0.019[d]

[1)] The different letters on the data in the column indicate significant differences at $P<0.05$.

Table S4. (Continued)

Sample	Malonic acid	Succinic acid	Lactic acid	Acetic acid	Fumaric acid
1	0.122±0.003^{bcde}[1)]	0.899±0.005^{jklm}	ND	ND	0.003±0.000^{de}
2	0.186±0.004^{bc}	1.327±0.080^{hijk}	ND	ND	0.003±0.000^{d}
3	0.213±0.001^{b}	1.601±0.025^{ghij}	ND	ND	0.001±0.000^{h}
4	0.189±0.008^{bc}	1.838±0.014^{fghi}	ND	ND	0.005±0.000^{ab}
5	0.171±0.002^{bcd}	2.419±0.051^{de}f	ND	ND	0.001±0.000^{h}
6	0.072±0.001^{cde}	2.801±0.127^{cd}	ND	ND	0.003±0.000^{d}
7	0.083±0.004^{cde}	3.613±0.050^{ab}	ND	ND	0.002±0.000^{gh}
8	0.073±0.000^{cde}	2.671±0.087^{de}	ND	ND	0.002±0.000^{ef}
9	0.854±0.152^{a}	1.996±0.110^{efgh}	ND	ND	0.004±0.000^{bc}
10	0.058±0.015^{de}	1.256±0.048^{ijkl}	ND	ND	0.003±0.000^{d}
11	0.029±0.001^{e}	0.419±0.047^{m}	ND	ND	0.003±0.000^{d}
12	0.006±0.001^{e}	3.452±0.219^{abc}	ND	ND	0.002±0.000^{f}
13	0.047±0.003^{e}	0.622±0.029l^{m}	ND	ND	0.003±0.000^{d}
14	0.018±0.002^{e}	1.676±0.124^{ghi}	ND	ND	0.004±0.000^{c}
15	0.082±0.002^{cde}	0.663±0.060^{klm}	ND	ND	0.005±0.000^{a}
16	0.031±0.004^{e}	2.399±0.184^{def}	ND	ND	0.002±0.000^{h}
17	0.087±0.005^{cde}	3.443±0.202^{abc}	ND	ND	0.002±0.000^{fg}
18	0.036±0.002^{e}	0.639±0.006^{klm}	ND	ND	0.004±0.000^{ab}
19	0.037±0.002^{e}	3.982±0.120^{a}	ND	ND	0.003±0.000^{d}
20	0.048±0.004^{e}	1.712±0.598^{fghi}	ND	ND	0.003±0.000^{d}
21	0.017±0.001^{e}	2.676±0.496^{de}	ND	ND	0.002±0.000^{f}
22	0.009±0.001^{e}	3.017±0.078^{bcd}	ND	ND	0.002±0.000^{f}
23	0.032±0.002^{e}	0.832±0.098^{klm}	ND	ND	0.004±0.000^{c}
24	0.026±0.002^{e}	2.081±0.090^{efg}	ND	ND	0.002±0.000^{ef}
25	0.015±0.001^{e}	0.744±0.049^{klm}	ND	ND	0.003±0.000^{d}

[1)] The different letters on the data in the column indicate significant differences at P<0.05.

Table S5. Total amino acid content (g/100 g) of dried laver

Sample	Aspartic acid	Threonine	Serine	Glutamic acid
1	2.093±0.065$^{e1)}$	1.137±0.055^{fgh}	1.101±0.052^{fg}	2.426±0.137^{fgh}
2	2.007±0.024^{ef}	1.067±0.022^{fgh}	1.068±0.018^{fg}	2.384±0.029^{fgh}
3	2.055±0.026^{ef}	1.225±0.072^{fg}	1.125±0.036^{fg}	2.525±0.096^{f}
4	1.972±0.048^{ef}	1.146±0.021^{fgh}	1.112±0.019^{fg}	2.391±0.060^{fgh}
5	2.170±0.028^{e}	1.252±0.041^{ef}	1.149±0.034^{f}	2.483±0.000^{fg}
6	2.037±0.036^{ef}	1.018±0.042^{fgh}	1.002±0.031^{fgh}	2.588±0.093^{f}
7	1.678±0.022^{ef}	0.892±0.024^{gh}	0.862±0.014^{gh}	1.872±0.045^{gh}
8	1.598±0.076^{ef}	0.851±0.010^{h}	0.778±0.031^{h}	1.789±0.080^{h}
9	1.367±0.651^{f}	1.565±0.265^{de}	1.143±0.031^{f}	2.052±0.242^{fgh}
10	2.042±0.049^{ef}	1.217±0.025^{fg}	1.069±0.027^{fg}	2.430±0.019^{fgh}
11	3.137±0.007^{cd}	1.740±0.003^{cd}	1.731±0.002^{e}	3.786±0.023^{cde}
12	3.328±0.009^{bcd}	1.760±0.001^{cd}	1.753±0.000^{e}	3.861±0.018^{bcde}
13	3.476±0.047^{bcd}	1.962±0.049^{abc}	1.770±0.091^{e}	3.988±0.004^{abcde}
14	3.353±0.064^{bcd}	1.996±0.048^{abc}	1.838±0.029^{cde}	3.804±0.069^{cde}
15	3.799±0.001^{abc}	2.193±0.014^{ab}	2.063±0.031^{abcd}	4.316±0.065^{abc}
16	3.545±0.015^{abcd}	1.936±0.017^{abc}	1.903±0.008^{bcde}	3.826±0.027^{bcde}
17	4.209±0.003^{a}	2.239±0.008^{a}	2.219±0.004^{a}	4.559±0.026^{a}
18	3.449±0.014^{bcd}	1.968±0.019^{abc}	1.776±0.015^{e}	4.547±0.030^{a}
19	3.047±0.618^{d}	1.796±0.311^{cd}	1.664±0.321^{e}	3.584±0.726^{e}
20	3.353±0.042^{bcd}	1.861±0.012^{bcd}	1.808±0.025^{de}	3.867±0.057^{bcde}
21	3.092±0.021^{cd}	1.744±0.017^{cd}	1.685±0.003^{e}	3.523±0.030^{e}
22	3.656±0.021ab^{cd}	1.833±0.005^{cd}	1.873±0.019^{bcde}	4.227±0.098^{abcd}
23	3.903±0.049^{ab}	2.176±0.028^{ab}	2.092±0.020^{abc}	4.458±0.084^{ab}
24	3.929±0.024^{ab}	2.260±0.033^{a}	2.138±0.015^{ab}	4.239±0.061^{abcd}
25	3.314±0.028^{bcd}	2.045±0.011^{abc}	1.733±0.002^{e}	3.622±0.024^{de}

1) The different letters on the data in the column indicate significant differences at $P<0.05$.

Table S5. (Continued)

Sample	Proline	Glycine	Alanine	Cysteine
1	0.838±0.142^{bcde}[1)]	1.303±0.042^{gh}	2.397±0.099^{g}	0.088±0.026^{c}
2	0.842±0.117^{bcde}	1.242±0.008^{gh}	2.410±0.004^{g}	0.098±0.022^{c}
3	0.755±0.024^{cde}	1.330±0.048^{gh}	2.525±0.060^{g}	0.024±0.005^{c}
4	0.705±0.062^{de}	1.267±0.044^{gh}	2.318±0.071^{g}	0.053±0.007^{c}
5	0.764±0.013^{cde}	1.416±0.030^{fg}	2.479±0.017^{g}	0.049±0.001^{c}
6	0.454±0.167^{e}	1.150±0.025^{gh}	2.645±0.039^{g}	0.048±0.023^{c}
7	0.552±0.023^{e}	1.063±0.001^{gh}	2.341±0.003^{g}	0.039±0.005^{c}
8	0.440±0.025^{e}	0.975±0.017^{gh}	2.314±0.027^{g}	0.039±0.006^{c}
9	1.765±0.761^{a}	0.930±0.416^{h}	2.007±0.376^{g}	1.686±1.310^{a}
10	0.750±0.053^{cde}	1.374±0.001^{gh}	2.572±0.014^{g}	0.112±0.002^{c}
11	1.284±0.009^{abcd}	1.871±0.003^{ef}	4.891±0.004^{bcd}	0.279±0.012^{c}
12	1.341±0.005^{abcd}	1.965±0.000^{de}	5.644±0.002^{a}	0.317±0.001^{c}
13	1.477±0.060^{ab}	2.175±0.071^{bcde}	5.264±0.025^{abc}	0.484±0.026^{bc}
14	1.487±0.025^{ab}	2.210±0.033^{bcde}	4.753±0.169^{cde}	0.953±0.266^{abc}
15	1.654±0.005^{a}	2.415±0.012^{abcd}	4.831±0.039^{bcd}	1.409±0.017^{ab}
16	1.488±0.008^{ab}	2.136±0.013^{bcde}	4.362±0.009^{de}	0.820±0.020^{abc}
17	1.589±0.038^{a}	2.531±0.019^{ab}	5.217±0.045^{abc}	0.924±0.045^{abc}
18	1.362±0.066^{abcd}	2.182±0.007^{bcde}	5.461±0.113^{ab}	0.739±0.080^{abc}
19	1.220±0.264^{abcd}	1.947±0.399^{e}	3.605±0.738^{f}	0.618±0.094^{bc}
20	1.377±0.004^{abc}	2.078±0.018^{bcde}	4.638±0.137^{cde}	0.939±0.093^{abc}
21	1.248±0.013^{abcd}	1.930±0.010^{e}	5.023±0.037^{abcd}	0.485±0.004^{bc}
22	1.360±0.018^{abcd}	2.053±0.029^{cde}	4.706±0.028^{cde}	0.615±0.027^{bc}
23	1.592±0.016^{a}	2.474±0.030^{abc}	4.624±0.047c^{de}	0.477±0.031^{bc}
24	1.615±0.009^{a}	2.697±0.031^{a}	4.805±0.158^{bcde}	1.006±0.096^{abc}
25	1.372±0.001^{abc}	2.281±0.006^{abcde}	4.115±0.031^{ef}	0.871±0.027^{abc}

[1)] The different letters on the data in the column indicate significant differences at P<0.05.

Table S5. (Continued)

Sample	Valine	Isoleucine	Methionine	Tyrosine
1	0.436±0.039$^{d1)}$	0.572±0.026^{ab}	0.987±0.089^{abc}	0.727±0.113^{bc}
2	0.407±0.004^{d}	0.524±0.007^{ab}	0.897±0.001^{abc}	0.449±0.072^{bc}
3	0.390±0.012^{d}	0.805±0.062^{a}	1.306±0.024^{a}	ND
4	0.411±0.018^{d}	0.578±0.014^{ab}	0.996±0.019^{abc}	0.133±0.109^{c}
5	0.384±0.017^{d}	0.757±0.023^{a}	1.280±0.033^{a}	0.595±0.006^{bc}
6	0.340±0.015^{d}	0.729±0.056^{a}	1.162±0.006^{ab}	0.296±0.242^{bc}
7	0.279±0.000^{d}	0.575±0.024^{ab}	0.990±0.011^{abc}	0.456±0.003^{bc}
8	0.292±0.022^{d}	0.538±0.021^{ab}	0.924±0.001^{abc}	0.049±0.037^{c}
9	1.171±0.629^{c}	0.571±0.215^{ab}	0.724±0.494^{c}	1.012±0.826^{b}
10	0.367±0.008^{d}	0.391±0.319^{b}	1.220±0.014^{ab}	.633±0.031^{bc}
11	1.617±0.026^{abc}	0.656±0.003^{ab}	0.984±0.019^{abc}	2.288±0.020^{a}
12	1.650±0.032^{abc}	0.637±0.004^{ab}	1.005±0.023^{abc}	2.334±0.009^{a}
13	1.823±0.085^{ab}	0.727±0.036^{a}	1.143±0.051^{abc}	2.565±0.106^{a}
14	1.749±0.061^{ab}	0.660±0.002^{ab}	1.034±0.047^{abc}	2.470±0.058^{a}
15	2.108±0.025^{a}	0.853±0.029^{a}	1.293±0.003^{a}	2.877±0.010^{a}
16	1.772±0.008^{ab}	0.722±0.012^{a}	1.052±0.021^{abc}	2.486±0.019^{a}
17	1.952±0.073^{ab}	0.840±0.017^{a}	1.201±0.057^{ab}	2.867±0.048^{a}
18	1.762±0.003^{ab}	0.688±0.007^{ab}	1.148±0.021^{ab}	2.515±0.013^{a}
19	1.649±0.290^{abc}	0.657±0.131^{ab}	1.016±0.182^{abc}	2.226±0.434^{a}
20	1.642±0.007^{abc}	0.671±0.001^{ab}	0.985±0.008^{abc}	2.384±0.018^{a}
21	1.452±0.043^{bc}	0.611±0.006^{ab}	0.845±0.016^{bc}	2.157±0.022^{a}
22	1.620±0.030^{abc}	0.668±0.043^{ab}	0.951±0.020^{abc}	2.377±0.029^{a}
23	1.914±0.060^{ab}	0.778±0.028^{a}	1.152±0.038^{ab}	2.710±0.071^{a}
24	1.972±0.085^{ab}	0.735±0.007^{a}	1.136±0.059^{abc}	2.701±0.062^{a}
25	1.724±0.006^{ab}	0.661±0.019^{ab}	1.025±0.023^{abc}	.384±0.016^{a}

[1)] The different letters on the data in the column indicate significant differences at $P<0.05$.

Table S5. (Continued)

Sample	Phenylalanine	Histamine	Lysine	Arginine
1	0.781±0.058$^{f1)}$	0.264±0.014^{de}	0.993±0.031^{fg}	1.116±0.039^{ghi}
2	0.629±0.014^{fg}	0.222±0.001^{def}	0.941±0.018^{fg}	1.032±0.005^{ghi}
3	0.728±0.025^{fg}	0.069±0.003^{f}	1.114±0.031^{fg}	1.128±0.029^{gh}
4	0.691±0.016^{fg}	0.251±0.002^{def}	1.021±0.013^{fg}	1.134±0.025^{gh}
5	0.756±0.029^{fg}	0.264±0.009^{de}	1.139±0.013^{f}	1.336±0.093^{g}
6	0.650±0.022^{fg}	0.156±0.085^{def}	1.022±0.012^{fg}	1.085±0.076^{ghi}
7	0.578±0.012^{fg}	0.214±0.015^{def}	0.850±0.002^{g}	0.979±0.018^{hi}
8	0.571±0.009^{g}	0.105±0.026^{ef}	0.842±0.043^{g}	0.819±0.111^{i}
9	0.678±0.101^{fg}	0.493±0.181^{b}	1.135±0.007^{f}	1.263±0.007^{gh}
10	0.768±0.030^{fg}	0.287±0.002^{cd}	1.102±0.001^{fg}	1.240±0.008^{gh}
11	1.124±0.006^{e}	0.442±0.001^{bc}	1.704±0.019^{e}	1.879±0.010^{ef}
12	1.156±0.015^{de}	0.464±0.001^{b}	1.766±0.017^{cde}	1.961±0.004^{def}
13	1.351±0.002^{abcd}	0.479±0.024^{b}	1.873±0.043^{abcde}	2.060±0.090^{bcdef}
14	1.268±0.028^{bcde}	0.488±0.009^{b}	1.842±0.041^{abcde}	2.118±0.044^{bcde}
15	1.452±0.002^{ab}	0.548±0.005^{ab}	2.041±0.000^{ab}	2.315±0.008^{ab}
16	1.271±0.011^{bcde}	0.493±0.002^{b}	1.816±0.018^{bcde}	2.115±0.012^{bcde}
17	1.532±0.039^{a}	0.608±0.012^{ab}	2.114±0.069^{a}	2.471±0.014^{a}
18	1.276±0.003^{bcde}	0.698±0.009^{a}	1.853±0.003^{abcde}	1.966±0.006^{def}
19	1.146±0.212^{e}	0.476±0.095^{b}	1.639±0.320^{e}	1.801±0.346^{f}
20	1.218±0.014^{de}	0.483±0.004^{b}	1.781±0.019^{cde}	1.997±0.024^{cdef}
21	1.099±0.010^{e}	0.495±0.007^{b}	1.643±0.017^{e}	1.797±0.016^{f}
22	1.210±0.028^{de}	0.466±0.009^{b}	1.738±0.027^{de}	1.995±0.021^{cdef}
23	1.431±0.013^{abc}	0.567±0.006^{ab}	2.013±0.027^{abc}	2.240±0.034^{abcd}
24	1.445±0.023^{ab}	0.536±0.008^{ab}	1.974±0.044^{abcd}	2.299±0.022^{abc}
25	1.237±0.007^{cde}	0.448±0.017^{bc}	1.783±0.010^{bcde}	2.001±0.018^{cdef}

[1)] The different letters on the data in the column indicate significant differences at $P<0.05$.

Table S6. Cutting stress (kg·mm) of dried laver

Sample	Cutting stress
1	0.62±0.16$^{a1)}$
2	0.51±0.14^{a}
3	0.56±0.12^{a}
4	0.54±0.16^{a}
5	0.56±0.14^{a}
6	0.24±0.11^{a}
7	0.37±0.12^{a}
8	0.35±0.12^{a}
9	0.41±0.13^{a}
10	0.33±0.13^{a}
11	0.25±0.11^{a}
12	0.30±0.10^{a}
13	0.26±0.11^{a}
14	0.25±0.05^{a}
15	0.31±0.06^{a}
16	0.39±0.10^{a}
17	0.17±0.09^{a}
18	0.18±0.07^{a}
19	0.29±0.08^{a}
20	0.33±0.12^{a}
21	0.26±0.08^{a}
22	0.33±0.14^{a}
23	0.31±0.10^{a}
24	0.38±0.11^{a}
25	0.35±0.09^{a}

[1)] The different letters on the data in the column indicate significant differences at $P<0.05$.

Table S7. Accuracy and R^2 value of PLS-DA class 1 in quality index

PLS-DA Class 1		TM[1)]		CV[2)]		PR [3)]	
		Accuracy	R^2	Accuracy	R^2	Accuracy	R^2
Proximate composition	Moisture	100%	95%	98%	79%	100%	90%
	Ash	100%	77%	97%	57%	100%	75%
	Crude protein	100%	85%	84%	40%	81%	70%
	Crude lipids	100%	82%	98%	57%	100%	77%
ATP-related compounds	ATP	100%	79%	99%	43%	100%	70%
	ADP	100%	89%	91%	62%	88%	73%
	AMP	100%	97%	100%	92%	100%	78%
	IMP	99%	80%	91%	32%	100%	67%
	GMP	97%	81%	87%	62%	100%	67%
Organic acid	Oxalic acid	100%	89%	90%	54%	100%	62%
	Citric acid	98%	86%	90%	63%	92%	79%
	Malic acid	99%	78%	85%	49%	90%	70%
	Maleic acid	100%	87%	94%	64%	100%	81%
	Malonic acid	100%	62%	73%	21%	100%	83%
	Succinic acid	99%	83%	93%	49%	100%	65%
	Fumaric acid	99%	78%	82%	33%	95%	40%
Amino acids	Aspartic acid	100%	94%	99%	79%	100%	95%
	Threonine	100%	86%	98%	75%	97%	67%
	Serine	100%	94%	99%	79%	100%	95%
	Glutamic acid	100%	92%	97%	67%	95%	83%
	Proline	100%	88%	93%	68%	100%	69%
	Glycine	100%	89%	99%	77%	100%	86%
	Alanine	100%	97%	100%	85%	96%	60%
	Cysteine	100%	79%	86%	29%	100%	93%
	Valine	100%	92%	98%	73%	97%	79%
	Methionine	100%	88%	99%	69%	100%	76%
	Leucine	100%	93%	96%	73%	97%	86%
	Phenylalanine	98%	89%	94%	61%	100%	48%
	Histamine	100%	87%	99%	74%	100%	70%
	Lysine	100%	94%	99%	79%	100%	95%
	Arginine	100%	94%	99%	79%	100%	95%
	Tryptophan	100%	87%	95%	95%	100%	99%
Texture	Cutting stress	100%	94%	100%	91%	100%	80%

[1)] Trained model; [2)] Cross-validation model; [3)] Predict model; [4)] Root Mean Square Error.

Table S8. Accuracy and R^2 value of PLS-DA class 2 in quality index

PLS-DA Class 1		TM[1)]		CV[2)]		PR [3)]	
		Accuracy	R^2	Accuracy	R^2	Accuracy	R^2
Proximate composition	Moisture	100%	95%	98%	77%	100%	91%
	Ash	99%	79%	86%	55%	95%	67%
	Crude protein	99%	90%	93%	58%	92%	94%
	Crude lipids	100%	90%	92%	63%	100%	70%
ATP-related compounds	ATP	100%	90%	94%	67%	96%	71%
	ADP	100%	85%	93%	58%	93%	67%
	AMP	100%	93%	99%	77%	83%	98%
	IMP	99%	86%	89%	50%	88%	88%
	GMP	99%	77%	98%	52%	100%	80%
Organic acid	Oxalic acid	98%	82%	84%	47%	89%	73%
	Citric acid	99%	86%	86%	49%	93%	72%
	Malic acid	100%	81%	92%	51%	97%	82%
	Maleic acid	100%	90%	96%	70%	98%	85%
	Malonic acid	100%	91%	98%	82%	100%	74%
	Succinic acid	99%	88%	93%	63%	90%	79%
	Fumaric acid	96%	78%	82%	36%	80%	84%
Amino acids	Aspartic acid	100%	96%	100%	86%	100%	97%
	Threonine	99%	80%	92%	58%	94%	82%
	Serine	100%	96%	100%	86%	100%	97%
	Glutamic acid	100%	93%	94%	66%	100%	98%
	Proline	99%	82%	81%	50%	96%	79%
	Glycine	99%	89%	95%	70%	96%	93%
	Alanine	100%	88%	100%	59%	100%	98%
	Cysteine	100%	96%	99%	87%	100%	93%
	Valine	100%	87%	93%	58%	100%	93%
	Methionine	100%	88%	95%	59%	95%	64%
	Leucine	100%	85%	93%	58%	100%	81%
	Phenylalanine	100%	84%	94%	57%	90%	69%
	Histamine	98%	81%	86%	50%	93%	85%
	Lysine	100%	96%	100%	86%	100%	97%
	Arginine	100%	96%	100%	86%	100%	97%
	Tryptophan	100%	57%	98%	80%	100%	97%
Texture	Cutting stress	100%	87%	98%	75%	100%	85%

[1)] Trained model; [2)] Cross-validation model; [3)] Predict model; [4)] Root Mean Square Error.

Table S9. Accuracy and R^2 value of PLS-DA class 3 in quality index

PLS-DA Class 1		TM[1)]		CV[2)]		PR [3)]	
		Accuracy	R^2	Accuracy	R^2	Accuracy	R^2
Proximate composition	Moisture	100%	90%	95%	77%	100%	91%
	Ash	98%	78%	92%	56%	91%	0%
	Crude protein	100%	95%	100%	88%	100%	94%
	Crude lipids	96%	81%	78%	39%	96%	70%
ATP-related compounds	ATP	100%	90%	92%	63%	92%	71%
	ADP	100%	89%	97%	74%	93%	67%
	AMP	100%	99%	100%	97%	100%	98%
	IMP	100%	86%	100%	67%	100%	88%
	GMP	100%	88%	95%	75%	100%	80%
Organic acid	Oxalic acid	100%	84%	92%	64%	88%	73%
	Citric acid	100%	87%	91%	53%	97%	72%
	Malic acid	100%	90%	99%	82%	96%	82%
	Maleic acid	100%	90%	94%	69%	97%	85%
	Malonic acid	99%	84%	92%	67%	100%	74%
	Succinic acid	99%	89%	91%	71%	95%	79%
	Fumaric acid	100%	87%	93%	65%	100%	84%
Amino acids	Aspartic acid	100%	99%	100%	98%	100%	97%
	Threonine	99%	86%	94%	69%	100%	82%
	Serine	100%	99%	100%	98%	100%	97%
	Glutamic acid	100%	99%	100%	97%	100%	98%
	Proline	100%	87%	95%	67%	100%	79%
	Glycine	99%	93%	98%	81%	100%	93%
	Alanine	100%	99%	100%	97%	100%	98%
	Cysteine	100%	95%	95%	73%	100%	93%
	Valine	100%	96%	98%	83%	100%	93%
	Methionine	100%	73%	98%	30%	100%	64%
	Leucine	98%	90%	94%	69%	96%	81%
	Phenylalanine	100%	85%	87%	52%	96%	69%
	Histamine	100%	91%	97%	67%	100%	85%
	Lysine	100%	99%	100%	98%	100%	97%
	Arginine	100%	99%	100%	98%	100%	97%
	Tryptophan	100%	85%	100%	98%	100%	98%
Texture	Cutting stress	99%	90%	99%	82%	100%	85%

[1)] Trained model; [2)] Cross-validation model; [3)] Predict model; [4)] Root Mean Square Error.

Table S10. Classification performance of each regression model

Quality index			Moisture			Crude protein			Cutting stress		
			Accuracy	R^2	RMSE[4)]	Accuracy	R^2	RMSE	Accuracy	R^2	RMSE
PLS-DA	TM[1)]	Class 1	100.0	94.9	0.11	100.0	85.5	0.12	100.0	94.4	0.10
		Class 2	100.0	94.6	0.11	99.0	89.9	0.16	100.0	87.2	0.17
		Class 3	100.0	90.2	0.10	100.0	94.6	0.11	99.0	90.3	0.16
	CV[2)]	Class 1	97.9	78.9	0.24	84.1	39.8	0.27	100.0	90.5	0.12
		Class 2	97.5	77.2	0.24	92.9	58.4	0.34	97.7	75.4	0.23
		Class 3	95.3	77.3	0.16	100.0	87.6	0.17	99.0	82.1	0.21
	PR[3)]	Class 1	100.0	90.4	0.19	81.1	70.1	0.27	100.0	80.2	0.12
		Class 2	100.0	90.5	0.16	92.0	93.9	0.30	100.0	84.9	0.21
		Class 3	100.0	90.5	0.11	100.0	93.9	0.12	100.0	84.9	0.20
VIP-PLS-DA	TM	Class 1	99.0	90.4	0.16	100.0	86.9	0.12	100.0	94.4	0.10
		Class 2	100.0	90.8	0.15	98.0	88.4	0.17	100.0	90.6	0.14
		Class 3	99.4	76.0	0.16	98.6	89.7	0.15	100.0	90.0	0.16
	CV	Class 1	96.9	78.3	0.24	90.5	50.1	0.24	100.0	90.3	0.13
		Class 2	97.1	77.9	0.23	90.0	62.5	0.32	98.5	75.6	0.24
		Class 3	94.7	62.4	0.20	98.6	80.4	0.22	99.0	82.7	0.21
	PR	Class 1	100.0	85.3	0.17	81.1	39.0	0.29	100.0	76.1	0.14
		Class 2	100.0	85.9	0.20	92.0	53.6	0.41	100.0	82.5	0.22
		Class 3	100.0	80.3	0.15	100.0	89.8	0.15	100.0	84.3	0.20
ANN-DA	TM	Class 1	100.0	99.1	0.05	100.0	99.9	0.01	93.2	77.1	0.24
		Class 2	100.0	99.3	0.04	100.0	100.0	0.01	90.0	68.6	0.28
		Class 3	100.0	99.4	0.03	100.0	100.0	0.01	94.7	82.1	0.14
	CV	Class 1	100.0	95.9	0.00	99.4	79.3	0.17	81.2	52.2	0.44
		Class 2	99.2	93.3	0.10	100.0	86.4	0.19	75.8	38.8	0.47
		Class 3	100.0	93.6	0.10	100.0	97.6	0.08	90.0	68.2	0.20
	PR	Class 1	100.0	95.6	0.12	81.1	82.8	0.22	92.3	76.5	0.24
		Class 2	100.0	99.0	0.10	95.8	99.6	0.21	90.0	99.9	0.24
		Class 3	100.0	99.0	0.04	100.0	99.6	0.03	100.0	99.9	0.04
PLS-ANN-DA	TM	Class 1	100.0	99.9	0.01	100.0	99.1	0.03	98.8	83.0	0.17
		Class 2	100.0	99.8	0.02	100.0	98.6	0.06	72.1	37.3	0.37
		Class 3	100.0	99.8	0.02	100.0	99.3	0.04	82.3	50.3	0.35
	CV	Class 1	100.0	96.7	0.00	86.4	68.0	0.20	95.6	78.9	0.22
		Class 2	93.1	77.3	0.25	100.0	75.8	0.93	80.8	49.7	0.37
		Class 3	97.0	84.1	0.22	99.2	93.8	0.10	86.1	55.1	0.37
	PR	Class 1	100.0	96.9	0.08	81.1	72.4	0.28	100.0	83.6	0.10
		Class 2	100.0	98.9	0.09	92.0	97.4	0.27	94.1	71.2	0.20
		Class 3	100.0	98.9	0.06	100.0	97.4	0.08	88.1	71.2	0.28

[1)] Trained model; [2)] Cross validation model; [3)] Predict model; [4)] Root Mean Square Error.
